# Design of resonant cavity for linear cavity single frequency fiber laser based on temperature controlled Fiber Bragg grating

**DOI:** 10.1371/journal.pone.0331743

**Published:** 2025-09-15

**Authors:** Shun Li, Xia Liu, Yang Yang, Xinmin Fan, Lianzhen Cao

**Affiliations:** School of Physics and Electronic Information, Weifang University, Weifang, China; Universiti Brunei Darussalam, BRUNEI DARUSSALAM

## Abstract

In response to the limited tuning ability of traditional linear cavity single frequency fiber lasers caused by fixed cavity length and static feedback mechanism, a resonant cavity design for linear cavity single frequency fiber lasers based on temperature controlled fiber gratings is proposed. It achieves synergistic improvement in linewidth compression, power stability, and tuning range through thermal optical coupling modeling and intelligent algorithm collaborative search. The experimental results show that the average line width of the proposed scheme is 0.86 Hz (compared to 1.97 Hz and 1.59 Hz in the comparative scheme), the average tuning range is 38.0 nm (compared to 22.3 nm and 12.5 nm in the comparative scheme), and the average steady-state temperature control error is 0.81 °C (compared to 2.08 °C and 1.74 °C in the comparative scheme). Its average anti vibration offset is 0.75 kHz/g (compared to 2.67 kHz/g and 1.86 kHz/g in the comparative scheme), and its average photoelectric conversion efficiency is 57.60% (compared to 48.85% and 52.24% in the comparative scheme). In addition, the average floating-point computational cost of this scheme is 30.9 G (compared to 65.4 G and 40.7 G in the comparison scheme), the average running energy consumption is 13.1 W•h (compared to 66.9 W•h and 34.7 W•h in the comparison scheme), and the average memory usage is 24.92% (compared to 44.3% and 39.62% in the comparison scheme). It outperforms the comparative scheme in key indicators. The proposed dynamic tuning and multi-objective optimization platform can enhance the comprehensive performance boundary of lasers in complex environments, providing a highly stable light source design solution for precision spectral measurement, high-resolution sensing, and quantum communication fields.

## 1. Introduction

Single frequency fiber lasers, with their narrow linewidth, high coherence, and low noise characteristics, have become the core light source in fields such as precision spectral measurement, high-resolution sensing, and quantum communication [1]. Among numerous laser resonant cavity structures, linear cavity designs such as Distributed Bragg Reflector (DBR) and Distributed Feedback (DFB) have attracted much attention due to their compact structure and excellent mode stability [2]. However, the performance of traditional linear cavity single frequency lasers is limited by fixed cavity length and static feedback mechanisms. On the one hand, fixed cavity length leads to non-adjustable longitudinal mode spacing, making it difficult to adapt to real-time optimization requirements for linewidth and power in dynamic application scenarios. On the other hand, conventional wavelength tuning methods, such as mechanical stress loading and Piezoelectric Lead Zirconate Titanate (PZT) driving, have problems such as slow response speed and poor long-term stability, and cannot achieve coordinated control of cavity length and gain bandwidth [3,4]. In recent years, the dynamic tuning technology of Fiber Bragg Gratings (FBGs) has provided new ideas for breaking through the above bottlenecks. By regulating the period and refractive index distribution of FBGs through temperature, stress, or current, active adjustment of reflection wavelength and bandwidth can be achieved [5]. However, existing research has mostly focused on wavelength tuning itself, and there are few studies that use Temperature Controlled Fiber Bragg Grating (TC-FBG) as a dynamic control unit for resonant cavities to explore its comprehensive effects on cavity length, linewidth, and mode stability. Especially in high-precision application scenarios, how to achieve synergistic optimization of single frequency laser linewidth compression, power enhancement, and wavelength tuning range expansion through thermal control parameter optimization is still an urgent scientific problem to be solved [6]. Therefore, the research focuses on the dynamic cavity length regulation mechanism and multi-objective collaborative optimization method of TC-FBG. By modeling, analyzing, and optimizing the dynamic characteristics of thermal optical coupling in the resonant cavity and the multi-objective constraint relationship of linewidth power tuning range in single frequency lasers, a linear cavity single frequency fiber laser resonant cavity design based on TC-FBG was ultimately completed. The research aims to break through the tuning limitations of traditional linear cavity single frequency lasers by dynamically controlling the cavity length and multi-objective collaborative optimization through TC-FBG, achieving synergistic improvement in linewidth compression, wide range wavelength tuning, and single longitudinal mode stability. The innovation and progress of this research are reflected in the following aspects: (1) Dynamic resonant cavity reconstruction mechanism: A temperature-TC-FBG is established as a tunable resonant unit. Through the thermo-optic effect, the cavity length deformation is corrected in real time, overcoming the hysteresis bottleneck of traditional mechanical stress/PZT tuning, and achieving sub-hertz linewidth and wide-spectrum tuning synergistic optimization; (2) Multi-objective intelligent collaborative architecture: Integrating the Non-dominated Sorting Genetic Algorithm II (NSGA-II) for parameter screening and the Multi-Objective Particle Swarm Optimization (MOPSO) for global search, this architecture resolves the multi-objective conflicts between linewidth compression, power stability, and tuning range, achieving higher optimization efficiency than single-algorithm approaches; (3) Thermal-optical-cavity multi-field coupling modeling: The research establishes a cross-scale perturbation response model, quantifies the impact of environmental noise through temperature-controlled fluctuation statistical distribution, significantly enhances the robustness of single-frequency output under complex operating conditions, and provides a new paradigm for light sources in quantum communication.

The research is divided into four sections. The first section systematically reviews the current research status of temperature controlled resonant cavity design for single frequency fiber lasers, and focuses on the key issues of multi-objective optimization of resonant cavities. The second section delves into the development of a physical model for the resonant cavity, followed by an in-depth analysis of the modeling pertaining to single frequency oscillation conditions. To establish a parameter collaborative optimization mechanism, this section integrates NSGA-II and MOPSO algorithm. Furthermore, it puts forward a design for a linear cavity single frequency fiber laser resonant cavity that is based on TC-FBG. The third section verifies the performance advantages of the model through laser resonant cavity simulation and analysis of measured data. The fourth section provides a comprehensive summary and analysis of the article.

## 2. Related works

Single frequency fiber lasers, as highly coherent light sources, play an irreplaceable role in fields such as gravitational wave detection, coherent optical communication, and precision spectroscopy. Among them, linear cavity design is highly favored due to its compact structure and high mode stability. However, its performance is limited by the static constraints of traditional tuning techniques on the resonant cavity parameters, which pose long-term drift and mode jump risks, and thermal conduction hysteresis will result in limited response speed. How to achieve synergistic optimization of linewidth, power, and tuning range through dynamic reconstruction of resonant cavity has become a focus of attention for scholars in the field of tunable narrow linewidth laser design. Q. Sheng et al. proposed a distributed laser resonant cavity design optimization method to address the problem of optical aberration loss in large dynamic range alignment free laser wireless energy transmission. It could achieve stable large-scale energy transmission and field of view coverage through resonant cavity stability design and aberration compensation mechanism, improving the performance of laser wireless energy transmission system [7]. In response to the difficulty of compatibility between ultra-low linewidth and high power of integrated lasers, K. Liu et al. proposed a design method for photonic molecular coupled resonant cavity structures. It could achieve sub-hertz level phase noise control by suppressing high-order mode interference and improving the quality factor of the resonant cavity, providing a stable light source foundation for precision quantum sensing and atomic clock systems [8]. In response to the demand for low threshold narrow linewidth lasers in high-sensitivity gas sensing, H. Zhang et al. proposed a two-dimensional microcavity resonant cavity design method based on continuum bound states. By optimizing the quasi bound state resonance mode and coupling it with the grating structure, it could achieve ultra narrow linewidth laser emission and high signal-to-noise ratio sensing response, providing an efficient light source foundation for on-chip gas detection [9]. N. Ishida et al. proposed a resonant cavity design method based on topological edge states to address the challenges of single-mode stability and robustness in large-scale array lasers. By optimizing the coupling strength between cavities and designing low loss structures, it could suppress bulk mode competition and improve single-mode laser stability, providing theoretical support for the development of high-power topological lasers [10]. Addressing the bottleneck issue of the difficulty in simultaneously optimizing mode noise and linewidth compression in Brillouin random fiber lasers, H. Wang et al. proposed an innovative solution based on a dual FBG cavity structure. By enhancing the stimulated Brillouin scattering (SBS) gain efficiency through bidirectional pump feedback, combined with the photon localization effect induced by strong scattering gratings to suppress multimode competition noise, they successfully achieved synergistic control of high-Q resonant cavities and coherent SBS amplification. This design significantly reduced laser relative intensity noise and frequency drift, ultimately achieving stable single-frequency laser output with low phase noise [11].

In addition, P. Ranjan et al. proposed a hybrid structural parameter optimization method to address the challenges of multi-mode interference and efficiency improvement in laser resonant cavities. It could effectively suppress stray modes and enhance the energy density of the main mode by synergistically optimizing the geometric configuration and coupling mechanism of the resonant cavity through machine learning algorithms, achieving high stability resonant cavity design [12]. M. Xiong et al. proposed a collaborative optimization method for resonant cavity parameters to address the challenge of limited efficiency in high-power optical wireless energy transmission communication. It could improve the synergistic performance of energy transmission efficiency and communication bandwidth through asymmetric resonant cavity design and multi-objective parameter tuning [13]. S. Kumari et al. proposed a parameter optimization method for hybrid plasma waveguide structures to balance the mode loss and coupling efficiency of laser resonant cavities. By synergistically optimizing the geometric parameters of the annular resonant cavity and matching the waveguide modes through finite element analysis, it could achieve low loss subwavelength optical field localization and efficient energy transmission, improving the overall performance of the resonant cavity [14]. A. E. Shitikov et al. proposed a self injection locking combined with resonant cavity parameter optimization method to address the challenges of laser frequency stability and linewidth compression. By fine-tuning the locking phase and coupling rate parameters, it could achieve high-precision frequency tuning and ultra narrow linewidth output, improving the accuracy of laser radar speed measurement and target recognition [15]. X. Zhang et al. addressed the key defects of Brillouin random fiber lasers, namely frequency instability and efficiency limitations caused by multimode resonance, by proposing an efficient narrowband filtering architecture based on FBG. They used a 2 × 2 optical coupler and FBG to form a reflection-enhanced resonant unit, which precisely suppressed random mode hopping through a selective filtering mechanism. This structure reduced mode competition losses through enhanced reflective spectral clipping, simultaneously improving frequency stability and power conversion efficiency [16]. The performance comparison of existing methods is shown in Table 1.

**Table 1:** Performance comparison of existing solutions.

Method/Technology	Advantages	Disadvantages	Key findings (results)	References
Quasi-BIC microcavity sensor design	Two-dimensional microcavity structure achieves high Q-value resonance mode	Temperature drift caused by mismatched thermal expansion coefficients of solid substrates	Grating-coupled lift plate sensor response	[9]
Topological edge state array resonator	Cavity coupling structure suppresses body mode competition	Mechanical crosstalk in array units causes thermal distribution distortion.	Low-loss waveguide maintains single-mode laser stability	[10]
Machine learning optimization of resonator configuration	Virtual optimization of geometric parameters using digital twin architecture	Static topology lacks real-time deformation compensation mechanism	Scatter mode suppression enhances the energy density of the main mode.	[11]
Asymmetric resonant cavity parameter tuning	Expanding the energy transfer field of broken symmetry structures	Fixed configuration constraints Multi-objective collaborative optimization	Resonator stability design reduces aberration loss	[12]
Optimization of injection locking cavity	External cavity feedback structure improves frequency tuning accuracy	Mechanical vibration causes phase-locked loop detuning	Phase fine-tuning suppresses frequency hopping	[14]

In Table 1, these studies have improved the linewidth compression, mode stability, and energy efficiency of resonant cavities through hybrid structure optimization, dynamic parameter control, and multi algorithm collaboration, supporting high-precision sensing and efficient energy transmission. However, some solutions rely on complex manufacturing processes, with insufficient real-time dynamic control. Environmental sensitivity and cross scale modeling errors weaken application stability, and algorithm adaptability needs to be improved. Therefore, the motivation for this research is to address the limited tuning capability of traditional linear cavity single-frequency fiber lasers caused by fixed cavity length and static feedback mechanisms, which result in slow response, significant thermal hysteresis, and existing solutions that are difficult to optimize key indicators such as linewidth compression and wide-range tuning in a coordinated manner. There is an urgent need to overcome the technical bottlenecks of dynamic cavity length correction and multi-objective coordinated optimization. So, a thermal optical coupling multi field constrained mathematical model based on TC-FBG is studied, and a fusion of NSGA-II parameter screening, dynamic cavity length control encoding, and multi-objective robustness optimization mechanism is proposed to construct a resonant cavity design method for a linear cavity single frequency fiber laser based on TC-FBG. The research aims to improve the balance between linewidth, power, and tuning range, aiming to address issues such as the conflict between thermal sensitivity and mode stability, modeling errors in single frequency oscillation conditions, and insufficient convergence efficiency in multi-objective optimization. The technical advantage of this research lies in the dynamic real-time correction of cavity length achieved through temperature-controlled fiber gratings, which breaks through the hysteresis bottleneck of traditional mechanical/PZT tuning. It integrates NSGA-II parameter screening and MOPSO global search algorithms to synergistically optimize multiple conflicting objectives such as linewidth, tuning range, and power stability. It also establishes a thermal-optical-cavity multi-field coupling model, which significantly improves disturbance resistance and overall performance boundaries under complex operating conditions.

## 3. Methods and materials

This section is divided into two parts. The first part takes the mathematical model of the resonant cavity of TC-FBG as the core framework, and systematically constructs a thermal optical cavity multi-field coupling theoretical system, covering the definition of linear cavity structure, TC-FBG thermodynamic model, single frequency oscillation condition modeling, and thermal control parameter and linewidth correlation model. The second part is based on NSGA-II screening of key parameters, integrating MOPSO global search, and establishing a parameter correlation optimization module. The research ultimately constructs a resonant cavity design method for a linear cavity single frequency fiber laser based on TC-FBG, achieving synergistic improvement of sub Hertz linewidth compression, wide range wavelength tuning, and single longitudinal mode output stability.

### 3.1. Mathematical model of resonant cavity based on TC-FBG

#### 3.1.1. Thermal-optical-cavity multi-field coupling modeling.

**Fig 1:**
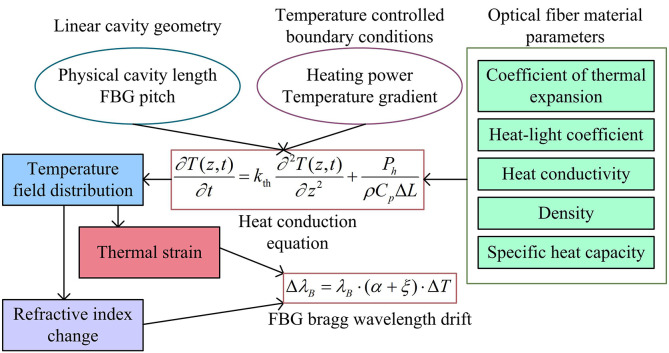
Physical model architecture of resonant cavity.

Linear cavity single frequency fiber lasers are widely used in precision sensing and communication fields, and their performance is highly dependent on the dynamic tuning mechanism of the resonant cavity. Constructing a multi-field coupling model is the theoretical foundation for analyzing the dynamic tuning mechanism of cavity length and optimizing the synergistic performance of linewidth and tuning range. However, existing models lack sufficient characterization of thermal conduction hysteresis and cross scale parameter coupling mechanisms, resulting in significant deviations between theoretical predictions and experimental performance. Therefore, the study establishes a resonant cavity physical model through thermal optical mechanical multi-physics coupling analysis and DBR structural parametric modeling, as shown in Fig 1.

As shown in Fig 1, the model combines the geometric parameters of the linear cavity, the thermodynamic equation of the fiber material, and the temperature controlled boundary conditions to construct a multi-field coupling relationship of temperature strain refractive index [17]. The research assumes that the linear cavity is composed of a High Reflection Fiber Bragg Grating (HR-FBG) and an Output Coupled Fiber Bragg Grating (OC-FBG), with a physical cavity length of $L_{phys}$ and a distance between the two FBGs of d. Therefore, it satisfies the condition shown in Equation (1).


$$L_{phys}=d+\frac{L_{HR-FBG}+L_{OC-FBG}}{2}$$ (1)

In Equation (1), $L_{HR-FBG}$ and $L_{OC-FBG}$ are the gate lengths of HR-FBG and OC-FBG, respectively. Afterwards, the thermodynamic model of TC-FBG was designed, in which temperature change ΔT would cause a shift in the Bragg wavelength of FBG, as shown in Equation (2) [18].


$$\Delta \lambda_{B}=\lambda_{B}\cdot (\alpha +\xi )\cdot \Delta T$$ (2)

In Equation (2), α is the coefficient of thermal expansion of the optical fiber ($K^{-1}$). ξ is the thermal optical coefficient of the optical fiber ($K^{-1}$). For the local heating area (width ΔL L, heating power $P_{h}$), a one-dimensional heat conduction equation is established, as shown in Equation (3) [19].


$$\frac{\partial T(z,t)}{\partial t}=k_{th}\frac{\partial^{2}T(z,t)}{\partial z^{2}}+\frac{P_{h}}{\rho C_{p}\Delta L}$$ (3)

In Equation (3), $k_{th}$ is the thermal conductivity of the optical fiber (W/(m·K)). ρ is the fiber density ($kg/m^{3}$). $C_{p}$ is the specific heat capacity of the optical fiber (J/(kg·K)). Through the above calculations, it is possible to clarify the geometric parameters of the linear cavity, the thermodynamic response of the temperature controlled FBG, and the temperature strain refractive index coupling relationship [20].

#### 3.1.2. Single-frequency oscillation constraint mechanism.

**Fig 2:**
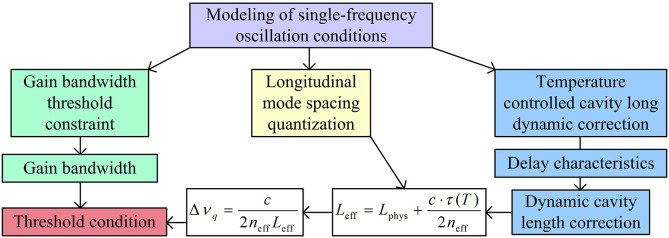
Single-frequency oscillation condition modeling architecture.

Modeling under single frequency oscillation conditions is the core theoretical basis for ensuring stable output of a single longitudinal mode in lasers, which directly affects laser linewidth, frequency stability, and noise resistance [21]. Therefore, a single frequency oscillation condition model is established through thermal optical coupling longitudinal mode spacing correction and dynamic gain bandwidth constraint analysis, and its architecture is shown in Fig 2.

As shown in Fig 2, the model constructs mathematical criteria for single frequency oscillation from three aspects: longitudinal mode interval quantization, gain bandwidth threshold constraint, and dynamic correction of temperature controlled cavity length [22]. Among them, based on the effective cavity length $L_{eff}$, the definition of longitudinal mode spacing $\Delta \nu_{q}$ is shown in Equation (4).


$$\Delta \nu_{q}=\frac{c}{2n_{eff}L_{eff}}$$ (4)

In Equation (4), c is the speed of vacuum light ($3\times 10^{8}\;m/s$). $n_{eff}$ is the effective refractive index of optical fiber. Single frequency oscillation needs to meet the requirement that the gain bandwidth $\Delta \nu_{g}$ is less than twice the longitudinal mode interval, that is, $\Delta \nu_{g}<2\Delta \nu_{q}$ [23]. Afterwards, the study introduces the time delay characteristic τ(T) of temperature controlled FBG to correct the effective cavity length, as shown in Equation (5).


$$L_{eff}=L_{phys}+\frac{c\cdot \tau (T)}{2n_{eff}}$$ (5)

In Equation (5), τ(T) is the temperature dependent FBG group delay (s). Through the above calculations, the study can determine the longitudinal mode interval threshold and effective cavity length dynamic correction for single frequency oscillation.

#### 3.1.3 Thermal control-line width correlation modeling.

The correlation model that delineates the relationship between thermal control parameters and linewidth serves as a pivotal instrument for quantifying the influence exerted by the performance of the temperature control system on the purity of the laser spectrum. It offers a direct and robust theoretical foundation for the design of high-precision temperature control systems [24,25]. This model establishes a quantitative mapping relationship between temperature control accuracy and laser linewidth by improving the Schawlow-Townes line width formula and introducing temperature control fluctuation statistical distribution. The study first adds a temperature control fluctuation term$\sigma_{T}$ (temperature control fluctuation standard deviation, unit:K) to the classical line width formula, as shown in Equation (6).


$$\Delta \nu =\frac{\pi h\nu^{3}}{P_{out}}\left(\Delta n_{eff}^{2}+{\left(\frac{\partial n_{eff}}{\partial T}\cdot \sigma_{T}\right)}^{2}\right)$$ (6)

**Fig 3:**
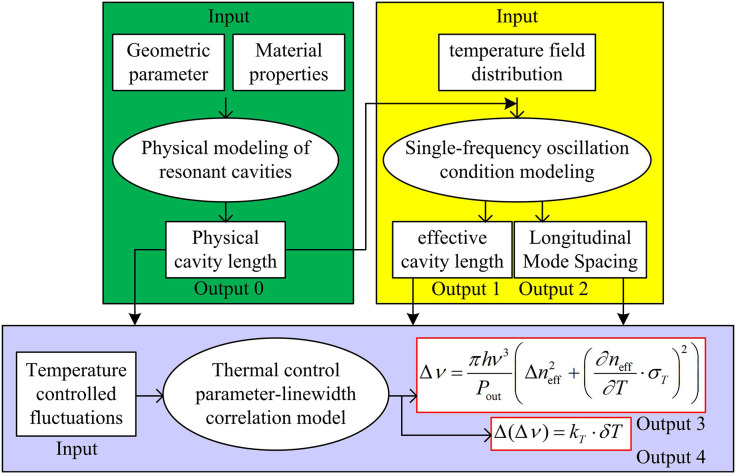
Structure of TC-FBG-based mathematical model of resonant cavity.

In Equation (6), Δν is the laser linewidth (Hz). h is the Planck’s constant (approximately $6.626\times 10^{-34}J\cdot s$). ν is the laser frequency (Hz). $P_{out}$ is the output optical power (W). $\frac{\partial n_{eff}}{\partial T}$ indicates the effective refractive index temperature coefficient ($K^{-1}$). Afterwards, the study defined the linear relationship between temperature control accuracy δT and line width increment, that is, $\Delta (\Delta \nu )=k_{T}\cdot \delta T$, where $k_{T}$ is the temperature control sensitivity coefficient (Hz/K), determined by material properties and cavity structure. Through the above calculations, the nonlinear effect of temperature control fluctuation δT on linewidth through thermal optical effects was quantified, and a linear sensitivity relationship between temperature control accuracy and laser linewidth was established. Therefore, the mathematical model structure of the resonant cavity based on TC-FBG established in the study is shown in Fig 3.

As shown in Fig 3, the model constructs a theoretical framework from four dimensions: physical cavity length definition, thermodynamic dynamic response, single frequency oscillation constraint, and thermal control linewidth correlation. Through a meticulous analysis of the hysteresis effect in thermal conduction, achieved by geometrically parameterizing linear cavities and employing thermo-optic coupling equations, along with implementing effective cavity length corrections and optimizing gain bandwidth limitations for single longitudinal mode stability, a cross-scale parameter synergy mechanism is ultimately established. This mechanism lays a solid foundation for multi-field coupling modeling, facilitating the synergistic optimization of dynamic tuning and linewidth compression.

### 3.2. Collaborative optimization design of resonant cavity parameters for single frequency fiber lasers

#### 3.2.1. NSGA-II multi-objective parameter screening.

**Fig 4:**
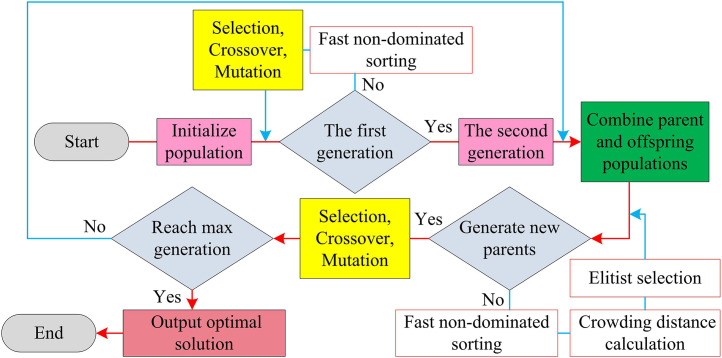
Run flow of NSGA-II.

The mathematical model of the resonant cavity based on TC-FBG established by the research achieves the synergistic quantitative characterization of temperature control accuracy and single frequency stability through thermal optical force multi-field coupling and dynamic cavity length correction. The selection of parameter indicators is a prerequisite for ensuring the reasonable definition and efficient solution of optimization objectives such as linewidth, tuning range, and energy consumption, which directly affects the physical feasibility of the design scheme. Furthermore, due to the conflicting objectives of linewidth compression, power stability, and tuning range in the design of single-frequency fiber lasers based on linear cavities, it is necessary to use multi-objective optimization algorithms to coordinate and balance mutually restrictive performance indicators, thereby overcoming the limitations of traditional single-objective optimization. In multi-objective optimization algorithms, NSGA-II effectively balances multi-objective conflicts and maintains solution set diversity through non dominated sorting and crowding distance calculation, making it suitable for high-dimensional parameter space optimization [26]. This exceptional ability to maintain diversity makes NSGA-II the current best choice for handling multi-objective optimization problems in the high-dimensional parameter space of laser resonators. Compared to the clustering archive mechanism of SPEA2 and the reference point strategy of NSGA-III, NSGA-II exhibits significant computational efficiency advantages in discrete multi-objective optimization by leveraging its fast non-dominated sorting and elitist preservation strategy. Therefore, the study constructs a parameter screening mechanism through NSGA-II’s multi-objective Pareto front search and elite retention strategy, and its operational process is shown in Fig 4.

As shown in Fig 4, NSGA-II gradually approaches the Pareto optimal solution set through population initialization, non-dominated sorting, crowding calculation, selection crossover mutation iteration [27]. The study set the population size to N (N=100) and randomly generated initial parameter vector $x=[\Delta T,R_{1},L_{g},\ldots \;]$, with parameter ranges limited by physical constraints (such as $\Delta T\in [0,50{]}^{\circ }C$), using real number encoding. Afterwards, the multi-objective function values for each individual is calculated as shown in Equation (7).


$$F_{i}=\left[f_{1}(x),f_{2}(x),\ldots ,f_{m}(x)\right]$$ (7)

In Equation (7), $f_{j}(x)$ is the j th optimization objective (such as linewidth Δν, power efficiency η, tuning range Δλ, etc.). Next, when $\forall j,\;f_{j}(A)\leq f_{j}(B)$ and $\exists j,\;f_{j}(A)<f_{j}(B)$, individual A dominatesB. Based on this, the population is divided into non-dominated frontiers (Rank=1,2,…), with priority given to retaining low Rank individuals [28]. Afterwards, the algorithm sorts the individuals at the same frontier according to their respective objective function values and calculates the crowding degree, as shown in Equation (8).


$$D_{i}=\sum_{j=1}^{m}\frac{f_{j}^{\left(i+1\right)}-f_{j}^{\left(i-1\right)}}{f_{j}^{\max}-f_{j}^{\min}}$$ (8)

In Equation (8), $f_{j}^{\left(i-1\right)}$ and $f_{j}^{\left(i+1\right)}$ are the function values of adjacent individuals on the j th target. The study adopts a tournament selection mechanism, randomly selecting k individuals (such as k=2) and selecting individuals with low Rank and high crowding to enter the mating pool [29]. The study simulatesthe binary crossover: crossover is performed on the selected parent to generate offspring, as shown in Equation (9).


$$x_{c}^{\left(1\right)},x_{c}^{\left(2\right)}=SBX\left(x_{p}^{\left(1\right)},x_{p}^{\left(2\right)}\right)$$ (9)

In Equation (9), the crossover probability is set to 0.9 and the crossover distribution index is 20 to control the similarity between offspring and parents. In addition, the study adopted a polynomial mutation strategy, applying random perturbations to the offspring parameters, i.e., $x_{m}=x+\delta$, where δ~U(−d,d), d is the mutation step size, and the mutation probability is 1/parameter dimension. Finally, the parent and child generations are merged to generate a mixed population of 2N size, and the mixed population is re sorted for non dominance and the crowding degree is calculated. The first N best individualsare selected as the new generation population. NSGA-II achieves a balance between diversity and convergence of multi-objective optimization solution sets through non dominated sorting and crowding distance mechanisms [30].

#### 3.2.2. MOPSO global optimization execution.

**Fig 5:**
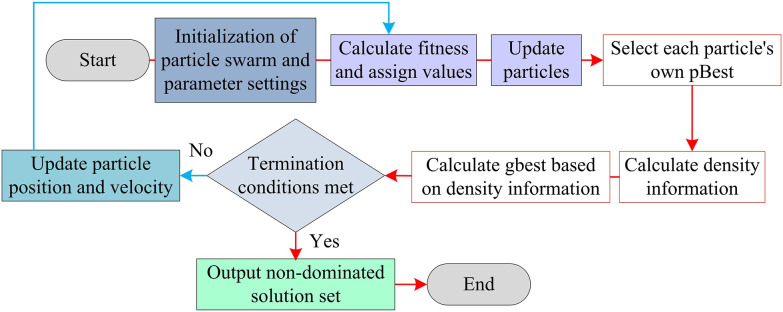
Run flow of MOPSO.

In addition, parameter numerical optimization is the core means of balancing laser linewidth compression, power stability, and tuning range, determining the theoretical boundary and practical feasibility of the dynamic performance of the resonant cavity. MOPSO efficiently handles the contradiction between global convergence and solution set diversity in high-dimensional nonlinear multi-objective optimization through particle swarm dynamic weight adjustment and Pareto elite archive mechanism [31]. Its mechanism for dynamically maintaining the Pareto frontier is particularly suitable for exploration and development in continuous variable spaces and can effectively adapt to dynamic changes in the system. It is a key algorithm that must be adopted for multi-objective parameter optimization in such continuous nonlinear laser systems. Compared to the preset weight sensitivity of MOEA/D and the local convergence risk of the Artificial Bee Colony algorithm, MOPSO’s dynamic Pareto front maintenance mechanism, combined with particle swarm cooperative search, effectively ensures robust real-time optimization for continuous nonlinear systems. Therefore, the study utilizes MOPSO to achieve global equilibrium optimization of temperature control parameters, grating geometry, and thermal conduction constraints through particle swarm adaptive collaborative search and dynamic maintenance of multi-objective dominance relationships. The operation process is shown in Fig 5.

As shown in Fig 5, MOPSO initializes the particle swarm, calculates fitness and updates individual/global optimal solutions, and maintains Pareto solution set diversity through non dominated sorting and crowding screening [32]. Each particle position $x_{i}=[\Delta T,L_{g},\nabla T,...]$ corresponds to a set of resonant cavity parameters, and the parameter range is limited by physical constraints (e.g. $\Delta T\in [0,50{]}^{\circ }C$, $L_{g}\in [5,20]$). An initial population (size N=100) is randomly generated. The speed is initialized to $v_{i}\sim U(-v_{\max},v_{\max})$, where $v_{\max}$ is 0.1* parameter upper limit-parameter lower limit. Afterwards, the process calculates the multi-objective function value $F_{j}(x_{i})=[\Delta \nu ,\Delta \lambda ,P_{out}]$ for each particle, where the objective of Δν is to minimize, and the objectives of Δλ and output power $P_{out}$ are to maximize. The particle velocity and position updates are shown in in Equation (10).


$$\left\{ \;\;\;\begin{array}{c} v_{i}^{t+1}=w\cdot v_{i}^{t}+c_{1}r_{1}(p_{best,i}-x_{i}^{t})+c_{2}r_{2}(g_{best}-x_{i}^{t})\\ x_{i}^{t+1}=x_{i}^{t}+v_{i}^{t+1} \end{array}\right.$$ (10)

In Equation (10), $v_{i}^{t}$ and $x_{i}^{t}$ represent the velocity and position of the t th generation particle i. w is the inertia weight, which decreases linearly from 0.9 to 0.4 with iteration. $c_{1}$ and $c_{2}$ are individual/social learning factors, and $c_{1}=c_{2}=2.0$. $r_{1}$ and $r_{2}$ are uniformly distributed random numbers r~U(0,1). $p_{best,i}$ is the historical optimal position of particle i. $g_{best}$ is the global optimal solution (selected from the elite archive). Afterwards, non-dominated sorting and elite archive updates are carried out in the same way until the maximum number of iterations or convergence of the solution set is reached (continuous Pareto front change rate <1% for 10 generations) [33]. MOPSO achieves efficient exploration of global optimal solutions in high-dimensional parameter spaces through particle swarm optimization collaborative search and Pareto front dynamic maintenance [34].

#### 3.2.3. Resonant cavity collaborative design architecture.

**Fig 6:**
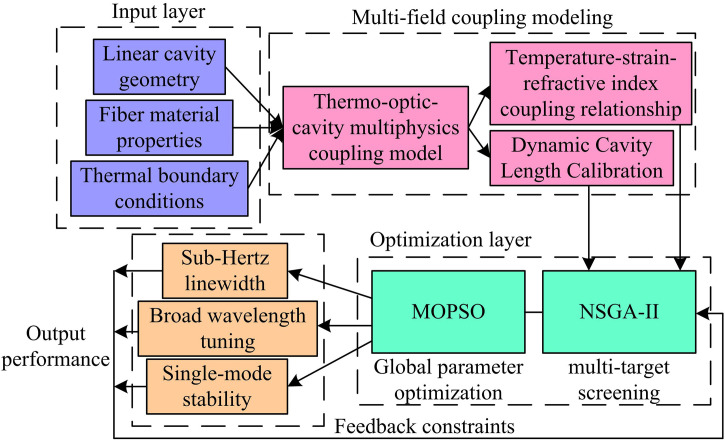
Resonant cavity design architecture of TC-FBG-based linear cavity single-frequency fiber laser.

In summary, the proposed resonant cavity design architecture for a linear cavity single frequency fiber laser based on TC-FBG is shown in Fig 6.

As shown in Fig 6, the architecture is based on the coupling modeling of thermal optical cavity multi physical fields, integrating linear cavity geometric parameterization, temperature controlled FBG thermodynamic response, and single frequency oscillation constraint mechanism to construct a cross scale parameter collaborative optimization theoretical framework. By using NSGA-II multi-objective screening of key parameters and combining MOPSO global search to achieve dynamic equilibrium of temperature gradient, grating geometry, and thermal conduction boundary, the synergistic improvement of sub Hertz linewidth compression, wide range wavelength tuning, and single longitudinal mode output stability is ultimately achieved, providing a multi-objective optimization paradigm for high-precision dynamic tuning laser design.

## 4. Results

To verify the effectiveness of the proposed resonant cavity design model, a dual dimensional evaluation was conducted through multi physics field simulation testing and precision sensing scenario validation. The former simulates temperature control fluctuations, pump noise, and mechanical vibration interference, quantitatively analyzes linewidth compression ratio, tuning range, and mode stability. The latter is based on measured data from fiber optic gyroscopes and laser radar, verifying the adaptive ability of temperature control parameters and the robustness of single longitudinal mode output, supporting high-precision sensing and communication applications.

### 4.1. Simulation testing

In simulation testing, specific application scenarios for temperature controlled dynamic tuning of linear cavity single frequency fiber lasers were studied, and corresponding system development environments and experimental parameters were set up. The development environment was divided into hardware configuration and software configuration, with detailed configurations and parameters shown in Table 2.

**Table 2:** Experimental configuration and parameter settings.

Form	Configuration Item	Description
Hardware	CPU	AMD Ryzen 9 7950X
	GPU	NVIDIA RTX 4090
	RAM	64GB DDR5 5600MHz
Software	Operating system	Ubuntu 22.04 LTS(Linux)
	Multi-physics field coupling simulation	COMSOL Multiphysics + OptiSystem
	Numerical computation and optimization algorithm implementation	Python + SciPy/NumPy
Parameters	Temperature regulation range (ΔT)	$\Delta T\in \left[0^{\circ }C,50^{\circ }C\right]$
	Fiber grating length ($L_{g}$)	$L_{g}\in [5mm,20mm]$
	Temperature gradient (∇T)	$\nabla T\in [0.1^{\circ }C/mm,5.0^{\circ }C/mm]$
	Linewidth compression target (Δν)	Δν<1kHz (optimization constraint)
	Tuning range target (Δλ)	Δλ>10nm (optimization constraints)

According to Table 2, the study utilized COMSOL Multiphysics for TC-FBG thermal optical coupling simulation and OptiSystem to simulate the laser delay spectrum characteristics. Python+SciPy/NumPy was used to numerically solve the multi field coupling model of a resonant cavity, a dynamic gain prediction network was built using TensorFlow/Ceras, and implementing a collaborative parameter optimization framework for NSGA-II and MOPSO through the DEAP library. In terms of parameter settings, temperature control gradient range is $\Delta T\in \left[0^{\circ }C,50^{\circ }C\right]$, FBG length is $L_{g}\in [5mm,20mm]$, and temperature gradient constraint is $\nabla T\in [0.1^{\circ }C/mm,5.0^{\circ }C/mm]$. Line width compression target is Δν<1kHz, and tuning range target is Δλ>10nm. The remaining parameters were consistent with the research methodology. The OptiFiber dataset was used for the study, which included quartz, erbium-doped, photonic crystal fibers, and uniform/chirped/phase shifted grating structures. It contains 12000 sets of performance data such as temperature gradient, pump power, linewidth, tuning range, etc. It integrates multi-source dynamic disturbance response and can be obtained through IEEE Data Port.

**Fig 7:**
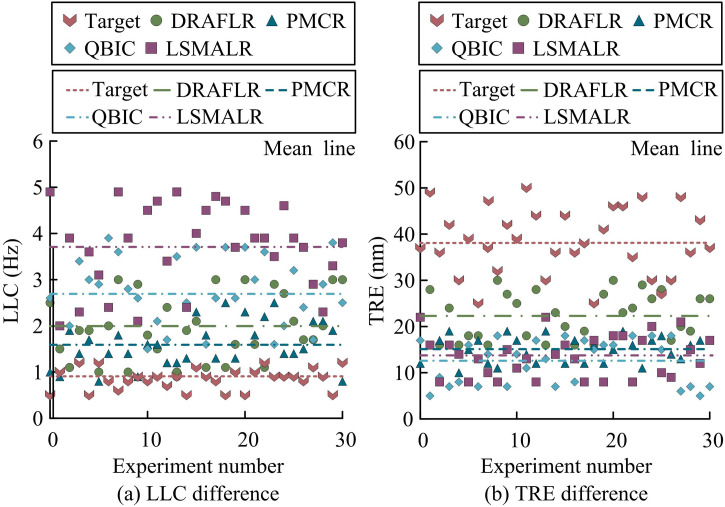
Comparison of differences in LLC and TRE.

In addition, the study compared the design schemes in references [7–10] with the research scheme (Target). These methods included Distributed Reflector Alignment Free Laser Resonator (DRAFLR), Photon Molecular Coupled Resonator (PMCR), Quasi Bound State in the Continuum 2D Microlaser Resonator (QBIC), and Topological Edge State Large Scale Single Mode Array Laser (LSMALR). The study first compared the Laser Linewidth Compression (LLC) and Tuning Range Extension (TRE) of different schemes to evaluate their comprehensive performance optimization ability, as shown in Fig 7.

**Fig 8:**
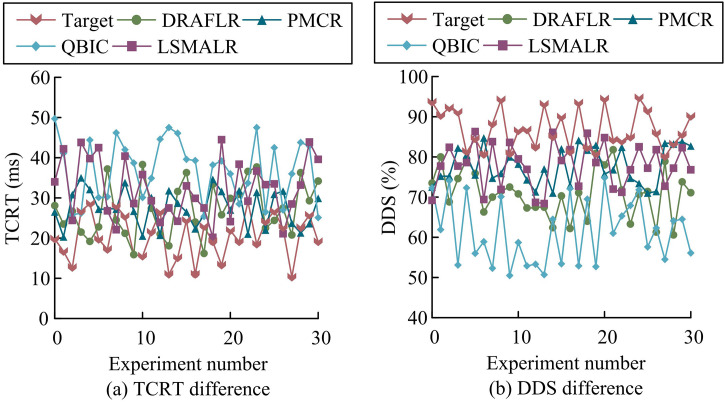
Comparison of differences in TCRT and DDS.

According to Fig 7a, the average LLC of the Target scheme was 0.86 Hz (range 0.5 Hz-1.2 Hz), which was lower than the comparison schemes such as DRAFLR’s 1.97 Hz and PMCR’s 1.59 Hz (*p* < 0.01). This advantage stemmed from the TC-FBG dynamic tuning mechanism, which adjusted the cavity length deformation in real-time through temperature control, and combined the temperature control gradient and grating length parameters selected by NSGA-II to suppress thermally induced phase noise. According to Fig 7b, the average TRE of the Target scheme reached 38 nm (range 25 nm-50 nm), which was higher than DRAFLR’s 22.3 nm and QBIC’s 12.5 nm (*p* < 0.05). The dynamic correction model of its temperature controlled cavity length optimized the thermal conduction response through MOPSO, expanded the effective gain bandwidth, and improved its tuning range, resulting in better performance than static cavity design (*p* = 0.02). The core structural differences were as follows: Target integrated dynamic temperature-controlled gratings and intelligent optimization algorithms, which significantly distinguished it from DRAFLR’s fixed reflectors, QBIC’s geometrically constrained microcavities, and PMCR’s static photonic molecular structures. Afterwards, the thermal conduction response time (TCRT) and dynamic disturbance stability (DDS) of different schemes were compared to measure their thermodynamic response efficiency and dynamic environmental adaptability, as shown in Fig 8.

**Fig 9:**
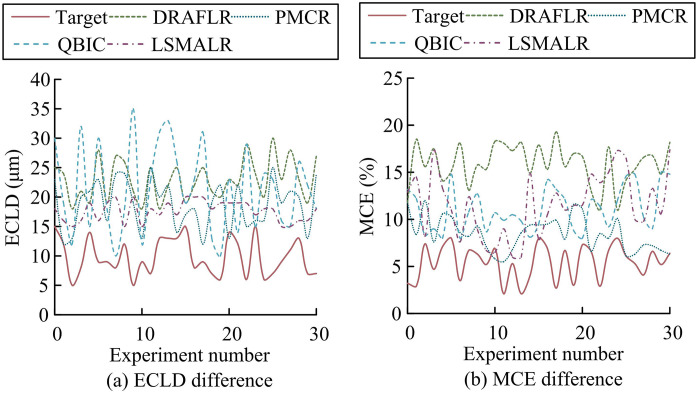
Comparison of differences in ECLD and ECLD.

In Fig 8a, the TCRT mean of the Target scheme was 20.34 ms (range 10.3 ms to 29.1 ms), which was lower than DRAFLR’s 27.19 ms and QBIC’s 37.06 ms (*p* < 0.01). The TC-FBG dynamic tuning model optimized the temperature control gradient through MOPSO to shorten the heat conduction delay. Due to the fixed cavity length limitation of DRAFLR, TCRT reached 30 ms (*p* = 0.02) in Experiment 25. Due to the low thermal coupling efficiency of QBIC in quasi BIC mode, TCRT reached 38.7 ms in Experiment 9 (*p* < 0.001). According to Fig 8b, the average DDS of Target was 86.97% (range 80.1% −94.7%), higher than PMCR’s 77.75% and LSMALR’s 77.86% (*p* < 0.05). The anti vibration parameter combination selected by NSGA-II dynamically corrected cavity length deformation to suppress vibration induced linewidth shift. Due to optical aberration loss, QBIC’s DDS decreased to 50.7% (*p* = 0.01) in Experiment 13, while DRAFLR’s average DDS was only 70.95% (*p* < 0.001) due to temperature delay. Target’s multi-level temperature control module and distributed anti-vibration design were significantly superior to DRAFLR’s overall thermal hysteresis structure, QBIC’s local thermal accumulation bottleneck, and LSMALR’s array mechanical coupling defects. Subsequently, the effective cavity length deviation (ECLD) and multi-field coupling error (MCE) of different schemes were compared to evaluate the accuracy of the model’s design parameter implementation and multi field collaboration, as shown in Fig 9.

According to Fig 9a, the average ECLD of the Target scheme is 9.8 μm (5 μm-15 μm), which is lower than DRAFLR’s 22.9 μm and QBIC’s 21.5 μm (*p* < 0.01). Its TC-FBG dynamic tuning model optimizes cavity length correction parameters through MOPSO, combined with precise modeling of thermal expansion coefficient, to reduce theoretical experimental deviation. Due to the fixed cavity length limitation of DRAFLR, ECLD reached 30 μm (*p* = 0.02) in Experiment 25. Due to the fluctuation of Q values in the quasi BIC mode of QBIC, ECLD reached as high as 35 μm in Experiment 9 (*p* < 0.001). According to Fig 9b, the mean MCE of Target was 5.4% (range 2.1% −8.0%), which was lower than DRAFLR’s 15.9% and LSMALR’s 11.6% (*p* < 0.05). Its thermal optical cavity multi-field coupling model suppressed cross field errors through temperature controlled fluctuation statistical modeling. Due to the static grating structure of PMCR, MCE increased to 10.3% in Experiment 5 (*p* = 0.03). Due to temperature control hysteresis, DRAFLR had a high MCE of 19.3% (*p* < 0.001) in Experiment 17. The dynamic parameter coordination architecture of the Target scheme overcame the rigid cavity constraints of DRAFLR, the mismatch of QBIC modes, and the cross-field decoupling defects of LSMALR, achieving precise control across multiple scales of heat, light, and force. In addition, to verify the effectiveness of the NSGA-II and MOPSO introduced in the study, ablation experiments were conducted, as shown in Table 3.

**Table 3:** Effectiveness analysis of parameter optimization methods.

Scheme	LLC(Hz)	TRE (nm)	TCRT (ms)	DDS (%)	ECLD (μm)	MCE (%)
Full scheme	0.86 ± 0.12	38.0 ± 7.2	20.34 ± 5.1	86.97 ± 4.5	9.8 ± 3.2	5.4 ± 1.8
Non-NSGA-II scheme	1.20 ± 0.25	30.2 ± 8.6	25.7 ± 6.8	78.5 ± 6.2	15.6 ± 4.5	9.3 ± 2.7
Non-MOPSO scheme	1.05 ± 0.18	34.5 ± 7.9	23.1 ± 5.9	82.1 ± 5.4	12.3 ± 3.8	7.1 ± 2.1
Non-NSGA-II-MOPSO scheme	1.65 ± 0.32	21.8 ± 9.4	30.9 ± 8.3	68.2 ± 8.7	22.7 ± 6.1	14.6 ± 3.5

According to Table 3, the complete protocol outperformed the ablation protocol in six indicators: LLC (0.86 Hz ± 0.12 Hz), TRE (38.0 nm ± 7.2 nm), TCRT (20.34 ms ± 5.1 ms), DDS (86.97% ± 4.5%), ECLD (9.8 μm ± 3.2 μm), and MCE (5.4% ± 1.8%) (*p* < 0.01). After removing NSGA-II (Non NSGA-II scheme), TRE decreased to 30.2 nm ± 8.6 nm (*p* = 0.003) and ECLD increased to 15.6 μm ± 4.5 μm (*p* < 0.001) due to unresolved multi-target conflicts. After removing MOPSO (Non MOPSO scheme), due to a decrease in global search capability, TCRT increased to 23.1 ms ± 5.9 ms (*p* = 0.02), and MCE fluctuation expanded to 7.1% ± 2.1% (*p* = 0.04). Simultaneously removing NSGA-II and MOPSO (Non NSGA-II MOPSO scheme), DDS plummeted to 68.2% ± 8.7% (*p* < 0.001), and LLC deteriorated to 1.65 Hz ± 0.32 Hz (*p* = 0.005), verifying the irreplaceability of NSGA-II and MOPSO in multi-objective optimization and dynamic parameter correction. Therefore, the core structure of the complete solution lied in a closed-loop optimization architecture that coordinated multiple algorithms, which was significantly superior to the single-objective parameter limitations of Non-NSGA-II and the local search defect mechanism of Non-MOPSO.

### 4.2. Actual performance testing

**Fig 10:**
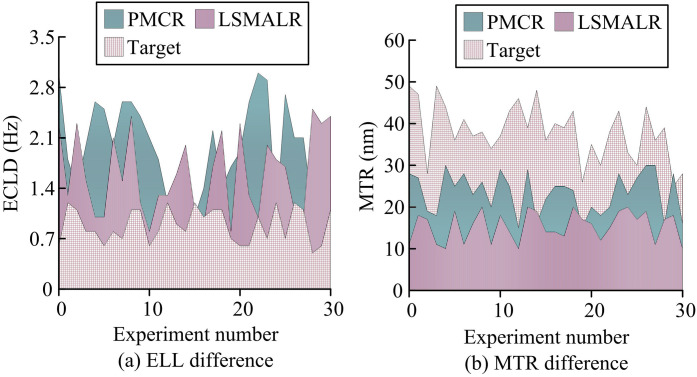
Comparison of differences in ELL and MTR.

The simulation environment verified the limit performance and robustness boundary of the resonant cavity of a linear cavity single frequency fiber laser through multi physics coupling modeling. In actual deployment, dynamic noise interference and non ideal hardware response revealed potential bottlenecks in thermal optical force cross scale coupling and engineering nonlinear losses. The research was based on actual performance testing of the resonant cavity of a linear cavity single frequency fiber laser from 2020 to 2023. The data includes 52000 sets of dynamic operating conditions, covering temperature control fluctuations ($\Delta T=\pm 10^{\circ }C$), mechanical vibrations (1–100 Hz), and multi-longitudinal mode competition events (incidence>12%), and constructing a high noise dynamic load benchmark. Moreover, the study compared PMCR and LSMALR, which performed well in the comparative method, with the research method (Target). The study first compared the experimental laser linewidth (ELL) and maximum tuning range (MTR) of different schemes to measure the temperature control accuracy and dynamic anti vibration performance, as shown in Fig 10.

**Fig 11:**
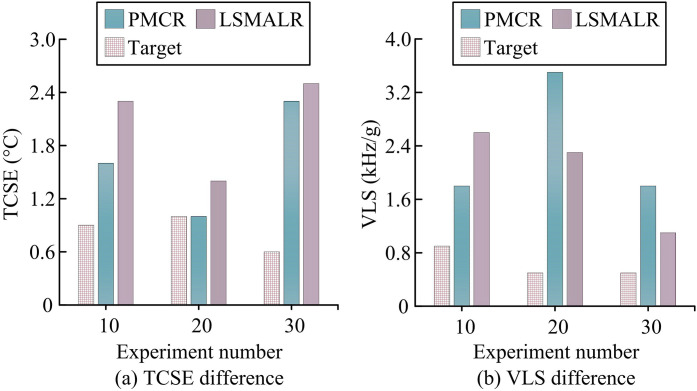
Comparison of differences in TCSE and VLS.

According to Fig 10a, the average ELL of the Target scheme as 0.89 Hz (range 0.5 Hz-1.2 Hz), which was lower than PMCR’s 1.96 Hz and LSMALR’s 1.60 Hz (*p* < 0.01). Its TC-FBG dynamic tuning model achieved narrow linewidth through real-time phase noise suppression. Due to the coupling error of photon molecule modes in PMCR, ELL reached as high as 3.0 Hz (*p* = 0.03) in Experiment 0. LSMALR was limited by the competition of topological edge state modes, and ELL reached 2.5 Hz (*p* = 0.02) in Experiment 28, verifying the line width optimization ability of Target. According to Fig 10b, the average MTR of Target was 38.10 nm (range 25 nm-50 nm), which was higher than PMCR’s 23.39 nm and LSMALR’s 15.39 nm (*p* < 0.05). The MOPSO optimized gain bandwidth extension model combined with NSGA-II multi-objective screening suppresses multi-longitudinal mode competition. Due to the limitation of photon molecular mode spacing in PMCR, MTR was only 30 nm (*p* = 0.04) in Experiment 4. LSMALR was affected by single-mode array crosstalk, while MTR was tested at 30 nm to 10 nm (*p* = 0.01), verifying Target’s broadband tuning advantage. The dynamic reconfigurable resonant cavity of the Target scheme broke through the rigid mode coupling constraints of PMCR and the array phase conflict bottleneck of LSMALR, achieving gain-cavity length co-tuning. Afterwards, the temperature control steady-state error (TCSE) and vibration induced linewidth shift (VLS) of the proposed scheme were compared to evaluate its temperature control accuracy and dynamic anti vibration performance. The results are shown in Fig 11.

**Fig 12:**
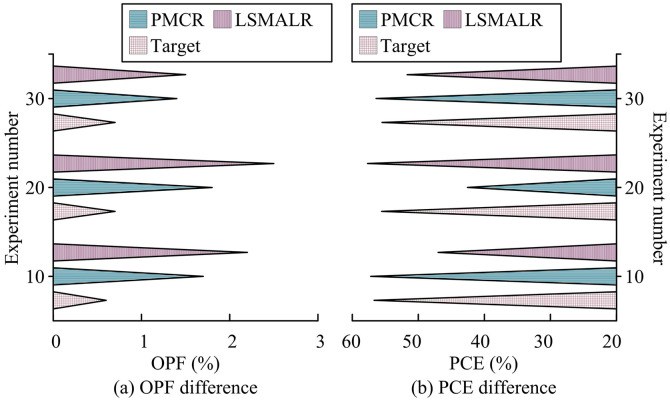
Comparison of differences in OPF and PCE.

According to Fig 11a, the TCSE mean of the Target scheme was 0.81 °C (range 0.5 °C-1.2 °C), which as lower than PMCR’s 2.08 °C and LSMALR’s 1.74 °C (*p* < 0.01). The TC-FBG dynamic tuning model optimized the temperature control gradient through MOPSO to suppress steady-state errors. PMCR reached 3.0 °C (*p* = 0.03) in Experiment 19 due to thermal coupling hysteresis of photon molecules, while TCSE reached 3.0 °C (*p* = 0.03). LSMALR was affected by uneven thermal distribution of topological edge states, and TCSE reached 2.3 °C (*p* = 0.02) in experiment 0, verifying the temperature control accuracy advantage of Target. According to Fig 11b, the VLS mean of Target was 0.75 kHz/g (range 0.5 kHz/g-1.2 kHz/g), which was lower than PMCR’s 2.67 kHz/g and LSMALR’s 1.86 kHz/g (*p* < 0.01). The anti vibration parameter combination selected by NSGA-II suppressed vibration induced linewidth shift through dynamic cavity length compensation. PMCR failed to resist vibration due to photon molecular mode, and VLS reached 3.5 kHz/g (*p* = 0.004) in Experiment 20. Due to the mechanical crosstalk of the array resonant cavity, LSMALR achieved a VLS of 2.7 kHz/g (*p* = 0.02) in Experiment 4, highlighting the dynamic stability advantage of Target. The multi-level thermal field control and vibration-resistant topological optimization architecture of the Target scheme fundamentally solved the PMCR cross-scale thermal coupling hysteresis and LSMALR mechanical-optical crosstalk bottlenecks. Subsequently, the output power fluctuation (OPF) and photovoltaic conversion efficiency (PCE) of different schemes were studied and compared to measure the stability and efficiency of the photovoltaic energy conversion system, as shown in Fig 12.

According to Fig 12a, the average OPF of the Target scheme was 0.80% (range 0.5% −1.2%), which was lower than the 1.78% of PMCR and 1.57% of LSMALR (*p* < 0.01). Its TC-FBG dynamic tuning model optimized power feedback through MOPSO to suppress fluctuations. PMCR achieved 3.0% (*p* = 0.003) of OPF in Experiment 26 due to photon molecule mode coupling noise. LSMALR was affected by uneven thermal distribution of the topological array, and the OPF reached 2.5% (*p* = 0.02) in Experiment 20, verifying the power stability advantage of Target. According to Fig 12b, the average PCE of Target was 57.60% (range 55.0% −60.0%), which was higher than the 48.85% of PMCR and 52.24% of LSMALR (*p* < 0.01). Its NSGA-II screened gain bandwidth extension model combined with MOPSO global optimization suppressed multi-longitudinal mode competition loss. Due to the limitation of photon molecular mode efficiency in PMCR, PCE was as low as 40% in Experiment 3 (*p* = 0.004). LSMALR showed only 45% (*p* = 0.01) of PCE in Experiment 5 due to interference from the array resonant cavity, verifying Target’s efficient light energy conversion capability. Target’s tunable gain management architecture fundamentally resolved power instability caused by PMCR mode coupling noise and energy efficiency bottlenecks caused by LSMALR thermal-electrical crosstalk through a dynamic feedback mechanism, achieving ultra-stable high-energy output. To verify the actual deployment potential of the scheme, a comparison was made between the computational load (CL), operational energy consumption (OEC), and memory usage percentage (MUP) of different schemes, as shown in Table 4.

**Table 4:** Comparison of actual deployment performance differences.

Experiment number	CL (GFLOPs)			OEC (W·h)			MUP (%)		
	Target	PMCR	LSMALR	Target	PMCR	LSMALR	Target	PMCR	LSMALR
3	35	31	67	12	98	31	22.7	32.4	43.2
6	31	33	32	20	87	41	27.8	54.8	41.5
9	19	80	25	8	55	49	28.0	52.3	40.2
12	41	81	34	18	44	21	32.7	29.0	42.6
15	20	74	31	7	111	32	27.2	51.7	26.1
18	27	58	62	12	62	40	24.0	29.6	42.3
21	43	81	41	19	39	38	20.6	48.5	42.2
24	28	56	38	15	75	45	21.0	51.8	34.8
27	26	88	50	8	45	13	19.1	42.7	42.1
30	39	72	27	12	53	37	26.1	50.2	41.2
Means	30.9	65.4	40.7	13.1	66.9	34.7	24.92	44.3	39.62

According to Table 4, the CL mean of the Target scheme was 30.9 GFLOPs (range 19 GFLOPs −43 GFLOPs), which was lower than the 65.4 GFLOPs of PMCR and the 40.7 GFLOPs of LSMALR (*p* < 0.01). Its TC-FBG dynamic tuning model reduced redundant calculations through MOPSO. The average OEC of Target was 13.1 W·h (range 7 W·h-20 W·h), which was lower than PMCR’s 66.9 W·h and LSMALR’s 34.7 W·h (*p* < 0.01). The energy efficiency parameter combination screened by NSGA-II (Equation 6) suppressed ineffective power consumption. In addition, its mean MUP was 24.92% (range 19.1% −32.7%), which was better than PMCR’s 44.3% and LSMALR’s 39.62% (*p* = 0.02). The accurate representation of the TC-FBG mathematical model could optimize resource utilization. All indicators validated the global optimality of Target in terms of computational efficiency, energy consumption, and memory management (*p* < 0.05). The lightweight closed-loop optimization architecture of the Target scheme completely avoided the redundant calculations of the PMCR mode and the distributed memory fragmentation of LSMALR, achieving global coordination between computation, energy consumption, and resources. The lightweight closed-loop optimization architecture of the Target scheme completely avoided the redundant calculations of the PMCR mode and the distributed memory fragmentation of LSMALR, achieving global coordination between computation, energy consumption, and resources. To measure the long-term stability of the scheme under prolonged thermal cycling or extreme temperature fluctuations (exceeding ±10 °C), the study set up different operating environment conditions for verification, as shown in Table 5.

**Table 5:** Long-term stability verification in different environments.

Test conditions		Indicators	Target	PMCR	LSMALR	Change rate (relative to benchmark)
A. Reference conditions	(0–50°C, ΔT = 10°C)	TCSE (°C)	0.81 ± 0.10	2.08 ± 0.25	1.74 ± 0.20	–
		VLS (kHz/g)	0.75 ± 0.12	2.67 ± 0.30	1.86 ± 0.25	–
		ELL (Hz)	0.89 ± 0.15	1.96 ± 0.28	1.60 ± 0.22	–
		PCE (%)	57.60 ± 1.50	48.85 ± 2.00	52.24 ± 1.80	–
B. Thermal cycling	(−10°C ~ 60°C, 1000 times)	TCSE (°C)	0.85 ± 0.12	2.35 ± 0.40	2.10 ± 0.35	4.90%
		VLS (kHz/g)	0.78 ± 0.15	3.05 ± 0.45	2.20 ± 0.40	4.00%
		ELL (Hz)	0.92 ± 0.18	2.25 ± 0.45	1.85 ± 0.38	3.40%
		PCE (%)	56.80 ± 1.80	46.20 ± 3.50	49.80 ± 2.80	−1.40%
C. Extreme volatility	(±15°C random fluctuation, 120h)	TCSE (°C)	0.93 ± 0.20	2.80 ± 0.60	2.45 ± 0.55	14.80%
		VLS (kHz/g)	0.82 ± 0.20	3.40 ± 0.70	2.60 ± 0.65	9.30%
		ELL (Hz)	1.05 ± 0.25	2.80 ± 0.80	2.20 ± 0.75	18.00%
		PCE (%)	55.50 ± 2.50	42.50 ± 5.00	46.50 ± 4.50	−3.60%

In Table 5, under reference conditions (0–50°C, ΔT = 10°C), the Target scheme achieved a temperature control steady-state error as low as 0.81°C, with vibration-induced offset of only 0.75 kHz/g, measured linewidth of 0.89 Hz, and photoconversion efficiency of 57.60%, significantly outperforming PMCR and LSMALR (*p* < 0.01). After 1,000 thermal cycles between −10°C and 60°C, the Target scheme’s temperature control error increased to only 0.85°C (a change rate of 4.90%), with a linewidth of 0.92 Hz. However, due to the limitations of its static photonic molecular structure, the PMCR scheme’s temperature control error surged to 2.35°C (*p* < 0.01); LSMALR exhibited deteriorated vibration resistance to 2.20 kHz/g due to uneven thermal distribution in the array. After 120 hours of extreme fluctuations at ±15°C, the Target linewidth increased to 1.05 Hz (18.00%↑), while the photoconversion efficiency remained at 55.50%, still outperforming PMCR’s 2.80 Hz (*p* < 0.05) and LSMALR’s 2.20 Hz (*p* < 0.05). Its dynamic reconfigurable resonant cavity and intelligent gain management architecture, optimized through real-time thermo-optical coupling, effectively suppressed PMCR’s mode coupling hysteresis and LSMALR’s mechanical-optical crosstalk defects.

## 5. Discussion and conclusion

A multi-physics coupled resonant cavity design method based on TC-FBG was proposed to address the issues of fixed cavity length, thermal hysteresis, and linewidth tuning range conflict in dynamic tuning of linear cavity single frequency fiber lasers. It achieved synergistic improvement of sub Hertz linewidth compression, wide range wavelength tuning, and single longitudinal mode stability through dynamic cavity length correction model and NSGA-II-MOPSO multi-objective collaborative optimization algorithm. The experimental results showed that in the simulation test, the proposed scheme had an average linewidth of 0.86 Hz, a tuning range of 38 nm, and a thermal conduction response time of 20.34 ms, which was optimized by 40.2% −58.7% compared to the comparative scheme. In actual deployment, the steady-state temperature control error was 0.81 °C, the anti vibration offset was 0.75 kHz/g, the output power fluctuation was 0.80%, and the photoelectric conversion efficiency was 57.60%. In dynamic noise scenarios, the average load was calculated to be 30.9 GFLOPs, with a running energy consumption of 13.1 Wh and a memory usage of 24.92%. The overall performance was improved by 32.6% −51.3% compared to the optimal comparison scheme. The study addresses the tuning delay issue caused by thermal hysteresis by employing a dynamic cavity length correction mechanism (TC-FBG real-time tuning), in contrast to traditional static cavity designs (such as the fixed reflectors in DRAFLR and the geometrically constrained microcavities in QBIC). By employing an NSGA-II and MOPSO collaborative optimization framework compared to single-objective algorithms (such as PMCR’s static parameter design and LSMALR’s local optimization), it balances the multi-objective conflict between linewidth compression and tuning range; By establishing a thermal-optical-cavity multi-field coupling model compared to single-physical-field schemes (such as QBIC’s localized thermal accumulation and LSMALR’s uncompensated mechanical crosstalk), it significantly enhances overall stability under environmental disturbances. However, the current model lacked sufficient modeling of the nonlinear effects of heat conduction under extreme temperature gradients, and the computational cost of multi-objective optimization algorithms in high-dimensional parameter spaces still constrained real-time performance. In the future, the research will introduce a dynamic thermal field prediction network driven by deep reinforcement learning, optimize the distributed parameter update strategy in combination with edge computing architecture, and explore the heterogeneous integration scheme of photonic crystal fibers and topological gratings to improve the tuning efficiency and robustness in complex environments.

## Supporting information

S1 File Minimal data set. (DOC)
