# Supplementary material for: Design of resonant cavity for linear cavity single frequency fiber laser based on temperature controlled Fiber Bragg grating
Source: PLoS One. 2025 Sep 15;20(9):e0331743. doi: 10.1371/journal.pone.0331743 (PMC12435697; doi:10.1371/journal.pone.0331743)
Supplement: S1 File — (DOC) [file pone.0331743.s001.doc]

**Data in Figure 7 (a)**

|  | Target | DRAFLR | PMCR | QBIC | LSMALR |
| --- | --- | --- | --- | --- | --- |
| 0 | 0.5 | 2.5 | 1 | 2.6 | 4.9 |
| 1 | 1 | 1.5 | 0.9 | 2 | 2 |
| 2 | 1.1 | 1.1 | 1.9 | 2 | 3.9 |
| 3 | 1.2 | 1.9 | 1.4 | 3.4 | 2.3 |
| 4 | 0.5 | 1.9 | 1.7 | 3 | 3.6 |
| 5 | 1.2 | 1 | 0.8 | 2.9 | 3.1 |
| 6 | 0.8 | 2 | 1.4 | 3.9 | 2.4 |
| 7 | 0.6 | 3 | 1.8 | 3.6 | 4.9 |
| 8 | 0.8 | 1 | 1.4 | 2.8 | 3.9 |
| 9 | 0.9 | 2.9 | 0.9 | 2.6 | 2.1 |
| 10 | 0.8 | 1.8 | 1.6 | 1.5 | 4.5 |
| 11 | 0.9 | 1.5 | 1.6 | 2.1 | 4.7 |
| 12 | 0.7 | 2.4 | 1.2 | 1.7 | 3.4 |
| 13 | 0.9 | 1 | 1.2 | 3.5 | 4.9 |
| 14 | 0.5 | 1.9 | 1.3 | 2.5 | 2.4 |
| 15 | 1.1 | 2.1 | 2.3 | 3.7 | 4 |
| 16 | 0.9 | 1.1 | 1.8 | 1.6 | 4.5 |
| 17 | 0.8 | 3 | 1.3 | 2.6 | 4.8 |
| 18 | 0.5 | 1.1 | 2.5 | 3.7 | 4.7 |
| 19 | 1 | 1.6 | 1.3 | 2.6 | 3.7 |
| 20 | 0.5 | 3 | 2.3 | 3.7 | 4.5 |
| 21 | 1 | 1.6 | 1.8 | 3 | 3.9 |
| 22 | 1.2 | 1.1 | 2.2 | 3.6 | 3.9 |
| 23 | 0.9 | 2.9 | 2.5 | 1.6 | 3.5 |
| 24 | 0.9 | 2.7 | 1.4 | 2 | 4.6 |
| 25 | 0.9 | 2.1 | 1.4 | 3.2 | 3.9 |
| 26 | 0.8 | 1.7 | 1.5 | 2.4 | 3.7 |
| 27 | 1.1 | 1.7 | 2.1 | 1.7 | 2.9 |
| 28 | 0.9 | 2 | 2.1 | 2.9 | 2.3 |
| 29 | 0.5 | 3 | 1.9 | 3.8 | 3.3 |
| 30 | 1.2 | 3 | 0.8 | 2.5 | 3.8 |
| means | 0.9 | 2.0 | 1.6 | 2.7 | 3.7 |

Data in Figure 7 (b )

|  | Target | DRAFLR | PMCR | QBIC | LSMALR |
| --- | --- | --- | --- | --- | --- |
| 0 | 37 | 22 | 12 | 17 | 22 |
| 1 | 49 | 28 | 16 | 5 | 16 |
| 2 | 36 | 16 | 17 | 9 | 8 |
| 3 | 42 | 24 | 19 | 7 | 16 |
| 4 | 30 | 16 | 10 | 8 | 14 |
| 5 | 39 | 18 | 15 | 16 | 8 |
| 6 | 25 | 18 | 17 | 7 | 13 |
| 7 | 37 | 16 | 12 | 14 | 10 |
| 8 | 32 | 30 | 11 | 18 | 8 |
| 9 | 42 | 27 | 19 | 7 | 15 |
| 10 | 39 | 25 | 17 | 14 | 11 |
| 11 | 50 | 18 | 14 | 11 | 13 |
| 12 | 44 | 28 | 12 | 17 | 8 |
| 13 | 30 | 16 | 19 | 13 | 22 |
| 14 | 36 | 23 | 14 | 7 | 14 |
| 15 | 44 | 20 | 12 | 18 | 16 |
| 16 | 36 | 16 | 17 | 8 | 13 |
| 17 | 38 | 19 | 12 | 17 | 8 |
| 18 | 25 | 16 | 16 | 15 | 17 |
| 19 | 41 | 27 | 12 | 16 | 8 |
| 20 | 46 | 30 | 15 | 16 | 18 |
| 21 | 46 | 23 | 19 | 18 | 18 |
| 22 | 35 | 24 | 16 | 18 | 8 |
| 23 | 48 | 29 | 11 | 17 | 17 |
| 24 | 30 | 26 | 17 | 18 | 20 |
| 25 | 27 | 28 | 18 | 15 | 10 |
| 26 | 30 | 17 | 14 | 18 | 9 |
| 27 | 48 | 20 | 13 | 6 | 21 |
| 28 | 36 | 19 | 16 | 7 | 15 |
| 29 | 43 | 26 | 17 | 5 | 12 |
| 30 | 37 | 26 | 17 | 7 | 17 |
| means | 38.0 | 22.3 | 15.0 | 12.5 | 13.7 |

**Data in Figure 8 (a**)

|  | Target | DRAFLR | PMCR | QBIC | LSMALR |
| --- | --- | --- | --- | --- | --- |
| 0 | 19.8 | 28.1 | 26.5 | 49.7 | 34 |
| 1 | 16.7 | 23.5 | 20.3 | 41.1 | 42.2 |
| 2 | 12.7 | 26.7 | 30.8 | 25.8 | 24.4 |
| 3 | 26.7 | 21.5 | 35 | 26.2 | 43.8 |
| 4 | 28.6 | 19.2 | 32.1 | 44.4 | 39.8 |
| 5 | 19.7 | 22.8 | 27.2 | 30.1 | 42.5 |
| 6 | 17.2 | 37.2 | 27.3 | 30.3 | 26.8 |
| 7 | 27.9 | 24.5 | 27.2 | 46.2 | 22.1 |
| 8 | 25.4 | 21.2 | 33.8 | 42 | 40.4 |
| 9 | 15.9 | 15.9 | 26.7 | 38.7 | 28.6 |
| 10 | 15.5 | 38.3 | 20.5 | 30.1 | 35.8 |
| 11 | 21.5 | 27.4 | 30.1 | 34.9 | 29.3 |
| 12 | 26.5 | 21 | 20.7 | 44.6 | 23.9 |
| 13 | 11 | 18.1 | 31.8 | 47.5 | 27.5 |
| 14 | 15.1 | 31.6 | 28.7 | 46.1 | 24.2 |
| 15 | 24.4 | 36.3 | 26.5 | 39.6 | 33 |
| 16 | 11 | 24 | 22.3 | 39.3 | 29.9 |
| 17 | 22.9 | 16.2 | 25.8 | 25.4 | 27.5 |
| 18 | 19.1 | 33.4 | 34.6 | 38.2 | 20.2 |
| 19 | 13.3 | 25.8 | 31.6 | 39.2 | 44.5 |
| 20 | 21.9 | 29.9 | 27 | 36 | 24.1 |
| 21 | 19.1 | 30.9 | 31.8 | 28.5 | 38.4 |
| 22 | 29.1 | 36.6 | 21 | 33.7 | 29.2 |
| 23 | 18.5 | 37.7 | 31.4 | 47.5 | 36.7 |
| 24 | 24.1 | 22.5 | 22 | 26.5 | 33.3 |
| 25 | 26.7 | 24.4 | 30.9 | 42.5 | 33.5 |
| 26 | 22.5 | 27.5 | 31.7 | 27 | 21.1 |
| 27 | 10.3 | 20.8 | 23.7 | 36 | 28.5 |
| 28 | 22.7 | 36.3 | 21.3 | 43.8 | 33.2 |
| 29 | 25.7 | 29.3 | 23.6 | 42.9 | 43.9 |
| 30 | 19.1 | 34.2 | 29.9 | 25.1 | 39.6 |

Data in Figure 8 (b)

|  | Target | DRAFLR | PMCR | QBIC | LSMALR |
| --- | --- | --- | --- | --- | --- |
| 0 | 93.6 | 73.5 | 72.5 | 72.3 | 69.2 |
| 1 | 90.3 | 80 | 75.2 | 61.9 | 77.7 |
| 2 | 92.2 | 68.8 | 75.2 | 74.1 | 82.4 |
| 3 | 91.1 | 74.6 | 82.2 | 53.1 | 77.7 |
| 4 | 81.1 | 79.5 | 80.5 | 72.3 | 78.6 |
| 5 | 84.5 | 75.9 | 75.5 | 56 | 86.3 |
| 6 | 80.6 | 66.3 | 84.7 | 58.9 | 69.4 |
| 7 | 88.2 | 70 | 74.7 | 52.3 | 83.8 |
| 8 | 94.2 | 71.5 | 75.9 | 70.1 | 71.9 |
| 9 | 80.9 | 72.5 | 80 | 50.5 | 83.6 |
| 10 | 86.5 | 70.8 | 78.2 | 58.7 | 79.5 |
| 11 | 86.7 | 67.3 | 74.3 | 52.9 | 76.9 |
| 12 | 82.4 | 67.5 | 71.3 | 53.3 | 68.7 |
| 13 | 93.1 | 67.5 | 77 | 50.7 | 68.4 |
| 14 | 85 | 62.4 | 71 | 64.5 | 86.1 |
| 15 | 89.9 | 70.3 | 82 | 53.4 | 79.1 |
| 16 | 81.4 | 62.2 | 72.9 | 72.1 | 81.8 |
| 17 | 93.4 | 71.1 | 84.1 | 52.9 | 72.7 |
| 18 | 82.2 | 64 | 81.7 | 69.5 | 85.9 |
| 19 | 80.8 | 80.9 | 82.9 | 52.7 | 78.6 |
| 20 | 94.4 | 78 | 75.7 | 74.8 | 84.8 |
| 21 | 84.2 | 81.8 | 76.8 | 61 | 72 |
| 22 | 83.6 | 71.8 | 82.4 | 65.3 | 71.2 |
| 23 | 84.9 | 63.3 | 74.7 | 67.9 | 76.8 |
| 24 | 94.7 | 70.7 | 73.4 | 71.8 | 82.5 |
| 25 | 91.5 | 71.4 | 71 | 57.6 | 77.2 |
| 26 | 85.9 | 61.3 | 71.4 | 62.3 | 81.8 |
| 27 | 80.1 | 78.9 | 83.4 | 54.5 | 72.7 |
| 28 | 83.2 | 60.6 | 83.2 | 64.1 | 77.2 |
| 29 | 85.5 | 73.8 | 83.8 | 64.5 | 82.3 |
| 30 | 90.1 | 71.1 | 82.7 | 56.1 | 76.8 |

Data in Figure 9 (a)

|  | Target | DRAFLR | PMCR | QBIC | LSMALR |
| --- | --- | --- | --- | --- | --- |
| 0 | 15 | 25 | 23 | 30 | 18 |
| 1 | 12 | 24 | 12 | 19 | 16 |
| 2 | 5 | 18 | 13 | 12 | 15 |
| 3 | 8 | 21 | 20 | 32 | 16 |
| 4 | 14 | 19 | 21 | 15 | 19 |
| 5 | 9 | 28 | 23 | 30 | 16 |
| 6 | 9 | 19 | 16 | 18 | 19 |
| 7 | 8 | 27 | 24 | 10 | 20 |
| 8 | 12 | 26 | 24 | 18 | 15 |
| 9 | 5 | 21 | 19 | 35 | 20 |
| 10 | 9 | 18 | 13 | 12 | 15 |
| 11 | 7 | 25 | 25 | 23 | 18 |
| 12 | 13 | 18 | 20 | 31 | 17 |
| 13 | 13 | 22 | 22 | 33 | 19 |
| 14 | 13 | 25 | 14 | 26 | 17 |
| 15 | 15 | 19 | 17 | 19 | 20 |
| 16 | 8 | 22 | 18 | 23 | 20 |
| 17 | 9 | 25 | 12 | 31 | 20 |
| 18 | 7 | 21 | 19 | 13 | 18 |
| 19 | 6 | 20 | 22 | 10 | 19 |
| 20 | 14 | 23 | 13 | 23 | 19 |
| 21 | 12 | 22 | 22 | 14 | 19 |
| 22 | 6 | 29 | 15 | 29 | 19 |
| 23 | 15 | 24 | 16 | 15 | 17 |
| 24 | 6 | 20 | 16 | 24 | 18 |
| 25 | 7 | 30 | 25 | 23 | 18 |
| 26 | 9 | 23 | 19 | 16 | 15 |
| 27 | 11 | 28 | 21 | 15 | 15 |
| 28 | 13 | 23 | 20 | 26 | 16 |
| 29 | 7 | 19 | 13 | 22 | 16 |
| 30 | 7 | 27 | 24 | 18 | 18 |

Data in Figure 9 (b)

|  | Target | DRAFLR | PMCR | QBIC | LSMALR |
| --- | --- | --- | --- | --- | --- |
| 0 | 3.2 | 11.8 | 11.7 | 13.2 | 12.9 |
| 1 | 2.9 | 18.5 | 8.4 | 12.2 | 14.5 |
| 2 | 7.4 | 15.6 | 12 | 8.3 | 8.2 |
| 3 | 4.7 | 17.5 | 7.6 | 9 | 17.3 |
| 4 | 7.1 | 14.1 | 10.6 | 8 | 13.3 |
| 5 | 8 | 15.1 | 10.3 | 14.8 | 11 |
| 6 | 3.5 | 18.1 | 8.6 | 8 | 7.6 |
| 7 | 6.7 | 13.1 | 8.2 | 10.8 | 12.4 |
| 8 | 6.3 | 15.8 | 9.3 | 12.7 | 8.9 |
| 9 | 5.2 | 15.3 | 7.1 | 8.7 | 6.7 |
| 10 | 6.9 | 18.3 | 5.8 | 10.7 | 6.5 |
| 11 | 2.1 | 18.1 | 5.5 | 9.9 | 9 |
| 12 | 5.3 | 17.3 | 6.8 | 10.5 | 6 |
| 13 | 2.1 | 18.2 | 8.6 | 9.8 | 6 |
| 14 | 4 | 14.9 | 9.4 | 8.1 | 14.9 |
| 15 | 8 | 17.9 | 9.4 | 9.5 | 7.9 |
| 16 | 6.9 | 15.3 | 9.5 | 14.2 | 10.5 |
| 17 | 2.7 | 19.3 | 9.9 | 13.2 | 12.8 |
| 18 | 6.7 | 15.6 | 7.9 | 12.1 | 11 |
| 19 | 3 | 17 | 11.6 | 8.8 | 11.5 |
| 20 | 7.3 | 16.7 | 11.1 | 8 | 11.5 |
| 21 | 6.6 | 13 | 6.6 | 12.1 | 14.8 |
| 22 | 2.9 | 11 | 8.5 | 11.4 | 13.9 |
| 23 | 6.7 | 17.7 | 8 | 9.2 | 15 |
| 24 | 8 | 11 | 10.1 | 12 | 17.3 |
| 25 | 6.1 | 14.2 | 6.1 | 14.6 | 16.5 |
| 26 | 5.3 | 15.3 | 6.3 | 14.8 | 10 |
| 27 | 4.1 | 16.6 | 7.3 | 10.2 | 9.8 |
| 28 | 6.6 | 16.8 | 7.2 | 9 | 13.3 |
| 29 | 5.2 | 14.8 | 6.7 | 15 | 10.6 |
| 30 | 6.4 | 18.2 | 6.3 | 14.8 | 17.4 |

Data in Figure 10 (a)

|  | PMCR | LSMALR | Target |
| --- | --- | --- | --- |
| 0 | 3 | 2.3 | 0.6 |
| 1 | 1.8 | 1.3 | 1.2 |
| 2 | 1.4 | 2.3 | 1.1 |
| 3 | 2 | 1.5 | 0.8 |
| 4 | 2.6 | 1 | 0.8 |
| 5 | 2.5 | 1 | 0.6 |
| 6 | 2 | 2.1 | 0.8 |
| 7 | 2.6 | 1.5 | 0.7 |
| 8 | 2.6 | 2.4 | 1.1 |
| 9 | 2.4 | 1.2 | 1.1 |
| 10 | 2.1 | 0.8 | 0.6 |
| 11 | 1.8 | 1.3 | 0.8 |
| 12 | 1.2 | 1.3 | 1.2 |
| 13 | 1 | 1.6 | 0.9 |
| 14 | 1.9 | 2 | 0.8 |
| 15 | 1 | 1.1 | 1.2 |
| 16 | 1.4 | 0.9 | 1 |
| 17 | 2.2 | 1.7 | 1.1 |
| 18 | 1.4 | 2.2 | 1.1 |
| 19 | 1.7 | 0.8 | 0.7 |
| 20 | 1.9 | 2.3 | 0.6 |
| 21 | 2.6 | 1.3 | 0.6 |
| 22 | 3 | 1 | 1 |
| 23 | 2.9 | 2 | 0.7 |
| 24 | 1 | 1.8 | 1.2 |
| 25 | 2.7 | 1.7 | 0.7 |
| 26 | 2.1 | 1.1 | 1.2 |
| 27 | 2.1 | 1 | 1.1 |
| 28 | 1.1 | 2.5 | 0.5 |
| 29 | 1.5 | 2.3 | 0.6 |
| 30 | 1.4 | 2.4 | 1.1 |

Data in Figure 10 (b)

|  | Target | PMCR | LSMALR |
| --- | --- | --- | --- |
| 0 | 49 | 28 | 11 |
| 1 | 47 | 27 | 18 |
| 2 | 28 | 19 | 17 |
| 3 | 49 | 18 | 11 |
| 4 | 44 | 30 | 10 |
| 5 | 36 | 25 | 19 |
| 6 | 41 | 28 | 11 |
| 7 | 37 | 23 | 16 |
| 8 | 38 | 26 | 20 |
| 9 | 34 | 20 | 11 |
| 10 | 37 | 29 | 18 |
| 11 | 43 | 25 | 14 |
| 12 | 46 | 15 | 10 |
| 13 | 39 | 29 | 20 |
| 14 | 48 | 16 | 19 |
| 15 | 36 | 22 | 14 |
| 16 | 40 | 25 | 14 |
| 17 | 39 | 25 | 13 |
| 18 | 43 | 24 | 20 |
| 19 | 26 | 15 | 17 |
| 20 | 35 | 20 | 16 |
| 21 | 30 | 18 | 12 |
| 22 | 38 | 20 | 15 |
| 23 | 43 | 28 | 19 |
| 24 | 33 | 23 | 20 |
| 25 | 30 | 27 | 17 |
| 26 | 44 | 30 | 19 |
| 27 | 36 | 30 | 11 |
| 28 | 39 | 16 | 17 |
| 29 | 25 | 28 | 18 |
| 30 | 28 | 16 | 10 |

**Data in Figure 11** (a)

|  | Target | PMCR | LSMALR |
| --- | --- | --- | --- |
| 10 | 0.9 | 1.6 | 2.3 |
| 20 | 1 | 1 | 1.4 |
| 30 | 0.6 | 2.3 | 2.5 |

**Data in Figure 11 (b**)

|  | Target | PMCR | LSMALR |
| --- | --- | --- | --- |
| 10 | 0.9 | 1.8 | 2.6 |
| 20 | 0.5 | 3.5 | 2.3 |
| 30 | 0.5 | 1.8 | 1.1 |

**Data in Figure 12** (a)

|  | Target | PMCR | LSMALR |
| --- | --- | --- | --- |
| 10 | 0.6 | 1.7 | 2.2 |
| 20 | 0.7 | 1.8 | 2.5 |
| 30 | 0.7 | 1.4 | 1.5 |

**Data in Figure 12** (b)

|  | Target | PMCR | LSMALR |
| --- | --- | --- | --- |
| 10 | 56.7 | 57.2 | 47 |
| 20 | 55.6 | 42.5 | 57.7 |
| 30 | 55.5 | 56.4 | 51.7 |
